# Addendum: A dispersive analysis of $\varvec{\eta '\rightarrow \pi ^+\pi ^-\gamma }$ and $\varvec{\eta '\rightarrow \ell ^+\ell ^-\gamma }$

**DOI:** 10.1140/epjc/s10052-022-11094-2

**Published:** 2022-12-21

**Authors:** Simon Holz, Christoph Hanhart, Martin Hoferichter, Bastian Kubis

**Affiliations:** 1 Albert Einstein Center for Fundamental Physics, Institute for Theoretical Physics, University of Bern, Sidlerstrasse 5, 3012 Bern, Switzerland; 2 Forschungszentrum Jülich, Institute for Advanced Simulation, Institut für Kernphysik, and Jülich Center for Hadron Physics, 52425 Jülich, Germany; 3 Helmholtz-Institut für Strahlen- und Kernphysik and Bethe Center for Theoretical Physics, Universität Bonn, 53115 Bonn, Germany

## Abstract

In this addendum to Ref. [1] we show that the mismatch between the $\rho$–$\omega$ mixing parameter $\epsilon _{\rho \omega }$ as extracted from $\eta '\rightarrow \pi ^+\pi ^-\gamma$ and $e^+e^-\rightarrow \pi ^+\pi ^-$ can be resolved by including higher orders in the expansion in $e^2$ in the description of the $\eta '\rightarrow \pi ^+\pi ^-\gamma$ decay. We repeat the analysis in this extended framework and update the numerical results accordingly.

**Addendum to: Eur. Phys. J. C** 10.1140/epjc/s10052-022-10247-7

## Extended formalism

Following the notation from Ref. [1] throughout, the spectrum for $P\rightarrow \pi ^+\pi ^-\gamma$ can be expressed as

1.1 $$\begin{aligned} \frac{\text {d}\varGamma (P\rightarrow \pi ^+\pi ^-\gamma )}{\text {d}s}&=16\pi \alpha \varGamma _0|F_\pi ^V(s)|^2 \bigg |P(s)\big (1+\varPi _\pi (s)\big )\nonumber \\&\quad - \frac{e^2 F_{P\gamma \gamma }}{s}-\frac{g_{P\omega \gamma }}{g_{\omega \gamma }}\frac{\epsilon _{\rho \omega }-e^2g_{\omega \gamma }^2}{M_\omega ^2-s-iM_\omega \varGamma _\omega }\bigg |^2, \end{aligned}$$

generalizing Eq. (D.14) in Ref. [1] by the next order in the expansion in $e^2$ (the sign convention is such that $g_{P\omega \gamma }<0$). The most important change, numerically, concerns $\epsilon _{\rho \omega }\rightarrow \epsilon _{\rho \omega }-e^2g_{\omega \gamma }^2$ in the numerator of the $\omega$ propagator, corresponding to the photon contribution in $\epsilon _{\rho \omega }$ as defined in resonance chiral perturbation theory [2–4]. In our formalism, $\epsilon _{\rho \omega }$, determined from a fit to the bare cross section for $e^+e^-\rightarrow \pi ^+\pi ^-$, does not include this VP effect, in line with the definition in Ref. [5] (numerically, it evaluates to $e^2g_{\omega \gamma }^2=0.34(1)\times 10^{-3}$). This shift removes the tension observed between $\eta '\rightarrow \pi ^+\pi ^-\gamma$ and $e^+e^-\rightarrow \pi ^+\pi ^-$ in Ref. [1].

The coefficients appearing in Eq. (3.9) of Ref. [1] are generalized according to Eq. (1.1):

1.2 $$\begin{aligned} \mathcal {A}_2&= - \varGamma (\eta ' \rightarrow \pi ^+ \pi ^- \gamma ) + 16 \pi \alpha \int _{4M_\pi ^2}^{M_{\eta ^\prime }^2} \text {d}s\, \varGamma _0 |F_\pi ^V(s)|^2\nonumber \\&\qquad \times \left| \frac{g_{\eta ' \omega \gamma }}{g_{\omega \gamma }}\frac{\epsilon _{\rho \omega }-e^2g_{\omega \gamma }^2}{M_\omega ^2-s-iM_\omega \varGamma _\omega } + \frac{e^2 F_{\eta ' \gamma \gamma }}{s} \right| ^2,\nonumber \\ \mathcal {A}_1&= 32 \pi \alpha \int _{4M_\pi ^2}^{M_{\eta ^\prime }^2} \text {d}s\, \varGamma _0 |F_\pi ^V(s)|^2\ \textrm{Re} \bigg [ P_\text {ev}(s)\big (1 + \varPi _\pi ^*(s)\big )\nonumber \\&\qquad \times \bigg (\frac{g_{\eta ' \omega \gamma }}{g_{\omega \gamma }}\frac{e^2g_{\omega \gamma }^2 - \epsilon _{\rho \omega }}{M_\omega ^2-s-iM_\omega \varGamma _\omega } - \frac{e^2 F_{\eta ' \gamma \gamma }}{s} \bigg ) \bigg ], \nonumber \\ \mathcal {A}_0&= 16 \pi \alpha \int _{4M_\pi ^2}^{M_{\eta ^\prime }^2} \text {d}s\, \varGamma _0 |F_\pi ^V(s)|^2 P_\text {ev}^2(s) \big |1 + \varPi _\pi (s) \big |^2. \end{aligned}$$

In the following, we provide the updated numerical results when including the additional $e^2$ effects as given in Eq. (1.1), implemented in the fit via Eq. (1.2).

**Table 1:** Comparison of the fit outcome of the differential decay width in Eq. (1.1) to the BESIII $\eta '\rightarrow \pi ^+ \pi ^- \gamma$ spectrum [6] of the binned maximum likelihood and minimum $\chi ^2$ strategies. The $\chi ^2/\text {dof}$ is 1.30 and 1.31, respectively, with the one of the Likelihood method extracted by means of the approximation described in App. C of Ref. [7]

Quantity	Likelihood	$\chi ^2$
$A \ [\,\text {GeV}^{-3}]$	17.12(35)	17.09(32)
$\beta \ [\,\text {GeV}^{-2}]$	0.714(55)	0.723(45)
$\gamma \ [\,\text {GeV}^{-4}]$	$-0.412(55)$	$-0.420(45)$
$\epsilon _{\rho \omega }\times 10^{3}$	1.998(67)	1.997(54)
$M_\omega \ [\,\text {MeV}]$	782.99(33)	783.00(27)

**Fig. 1:**
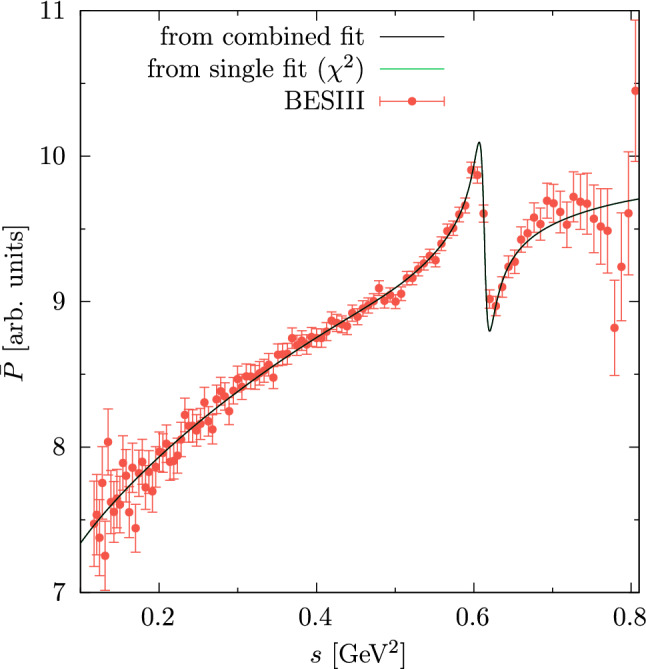
Fit to the differential decay rate of $\eta ' \rightarrow \pi ^+ \pi ^- \gamma$ (individually or combined with the VFF). To highlight potential differences in the $\rho$–$\omega$ region, we show the associated function $\bar{P}$, as defined in Eq. (3.11) of Ref. [1], compared to the experimental data from BESIII [6]. The two fits cannot be distinguished on this scale

**Table 2:**
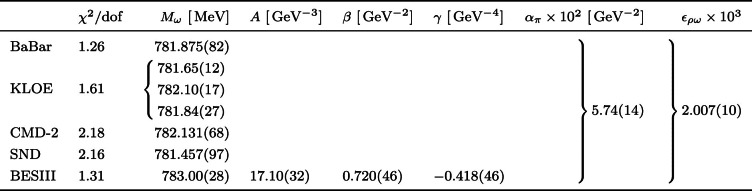
Combined fit to several pion VFF data sets (BaBar, KLOE, CMD-2, SND) and $\eta ' \rightarrow \pi ^+ \pi ^- \gamma$ spectrum (BESIII) with overall $\chi ^2/\text {dof} = 1.46$. In the row for KLOE, the three values for $M_\omega$ refer to the combinations of the global KLOE $\omega$ mass and the corresponding mass shifts of the three underlying data sets from 2008, 2010, 2012, respectively

## Numerical results

The updated fit parameters are collected in Table 1, Fig. 1, and Table 2. The main difference to the results presented in Ref. [1] is that the shift $\epsilon _{\rho \omega }\rightarrow \epsilon _{\rho \omega }-e^2g_{\omega \gamma }^2$ removes the tension between $e^+e^-\rightarrow \pi ^+\pi ^-$ and the $\eta '\rightarrow \pi ^+\pi ^-\gamma$ spectrum, markedly improving the quality of the combined fit.

The updated results for the TFF are shown in Fig. 2 and Table 3. In particular, the prediction for the slope parameter

2.1 $$\begin{aligned} b_{\eta '}=1.431(23)\,\text {GeV}^{-2} \end{aligned}$$

is reduced by about $1\sigma$, which traces back not to the change in $\epsilon _{\rho \omega }$ (which is marginal given the fact that the fit is dominated by $e^+e^-\rightarrow \pi ^+\pi ^-$), but to a stronger curvature in the polynomial *P*(*s*) (the coefficient $\gamma$ of the quadratic term increases by a factor 3).

**Fig. 2:**
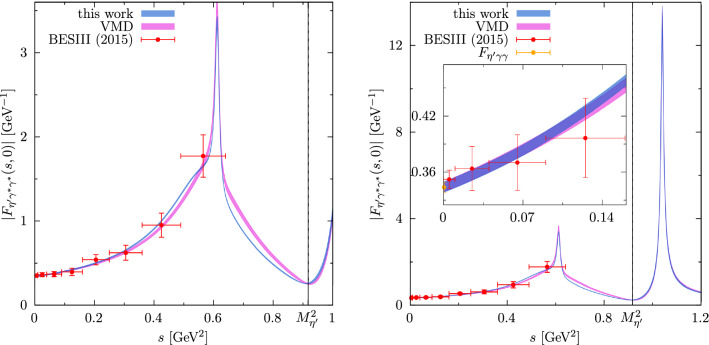
Determination of the $\eta '$ TFF in comparison to data from BESIII [8] (statistical and systematic errors added in quadrature) scaled with $F_{\eta ' \gamma \gamma }$ and the VMD model from Ref. [1] for the $\phi$ resonance; for the kinematic range accessible in $\eta '$ decays (left) and a larger time-like region including the $\phi$ resonance with inset magnifying the low-*s* region (right)

**Table 3:** Contributions from the various components of the TFF to the sum rules of the normalization and the slope parameter

	$(I=1)_{\epsilon _{\rho \omega }=0}$	$\varDelta (I=1)_{\epsilon _{\rho \omega }\ne 0}$	$(I=0)^\omega _{\epsilon _{\rho \omega }=0}$	$\varDelta (I=0)^\omega _{\epsilon _{\rho \omega }\ne 0}$	$(I=0)^\phi$	Total
Norm $[\%]$	69.18(86)	$-0.1388(19)$	7.06(22)	$-0.1397(47)$	15.85(61)	91.9(1.1)
$b_{\eta '} \ [\,\text {GeV}^{-2}]$	1.160(23)	0	0.1176(32)	0	0.1526(53)	1.431(23)
